# Structural Requirements for the Antifungal Activities of Natural Drimane Sesquiterpenes and Analogues, Supported by Conformational and Electronic Studies

**DOI:** 10.3390/molecules18022029

**Published:** 2013-02-05

**Authors:** Marcos Derita, Iván Montenegro, Francisco Garibotto, Ricardo D. Enriz, Mauricio Cuellar Fritis, Susana A. Zacchino

**Affiliations:** 1 Farmacognosia, Facultad de Ciencias Bioquímicas y Farmacéuticas, Universidad Nacional de Rosario, Suipacha 531, 2000 Rosario, Argentina; 2 Laboratorio de Ciencias Químicas y Recursos naturales, Facultad de Farmacia, Universidad de Valparaíso, Avenida Gran Bretaña 1111, Playa Ancha, 2340000 Valparaíso, Chile; 3 Departamento de Química, Facultad de Química, Bioquímica y Farmacia, Universidad Nacional de San Luis, Chacabuco 915, 5700 San Luis, Argentina; 4 IMIBIO-SL (CONICET), Chacabuco 915, 5700 San Luis, Argentina

**Keywords:** antifungal agents, drimanes, structure-activity relationships, stereo-electronic studies

## Abstract

Seventeen drimanes including polygodial (**1**), isopolygodial (**2**), drimenol (**3**) and confertifolin (**4**) obtained from natural sources and the semi-synthetic derivatives **5–17** obtained from **1–3**, were evaluated *in vitro* for antifungal properties against a unique panel of fungi with standardized procedures by using two end-points, MIC_100_ and MIC_50_. A SAR analysis of the whole series, supported by conformational and electronic studies, allowed us to show that the Δ^7,8^ -double bond would be one of the key structural features related to the antifungal activity. The MEPs obtained for active compounds exhibit a clear negative minimum value (deep red zone) in the vicinity of the Δ^7,8^ -double bond, which is not present in the inactive ones. Apart of this negative zone, a positive region (deep blue) appears in **1**, which is not observed either in its epimer **2** nor in the rest of the active compounds. The Log*P* of active compounds varies between 2.33 and 3.84, but differences in MICs are not correlated with concomitant variations in Log*P* values.

## 1. Introduction

**Figure 1:**
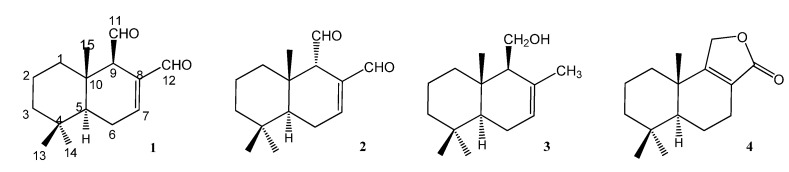
Drimanes isolated from *Polygonum acuminatum.*

In the course of our ongoing search for antifungal compounds from natural sources, we recently reported that the aerial parts of *Polygonum acuminatum* Kunth., used in the Argentinean traditional medicine for ailments related to fungal infections [1], possessed antifungal properties against yeasts and dermatophytes, these results supporting their ethnopharmacological use [2]. Polygodial (**1**, Figure 1) was isolated from the most active extract as the main compound responsible for the activity, although other drimane derivatives such as isopolygodial (**2**) drimenol (**3**) and confertifolin (**4**) (Figure 1) showed antifungal properties too [2].

Although the claims that **1** possesses antifungal activity were consistent in all previous reports [3,4,5,6], contradictory reports were found in the literature regarding the antifungal activities of its natural analogue **2**, which was variously reported to be either active or inactive, and based on this information, conflicting opinions about the main structural features required for drimanes to display antifungal activities were put forth. They can be summarized as follows: Fujita and Kubo stated that the β- orientation of the C-9 carbonyl group was indispensable for activity, since **1** (with an aldehyde in the C-9β orientation) but not **2** (with a C-9α aldehyde) possessed antifungal activity [7]. Conversely, Anke and Sterner [8] found that **2** did possess antifungal activity, although lower than that of **1**, which was corroborated by us in the paper cited above [2] and in a subsequent one [9]. Another structural feature which was assigned a role in the antifungal activity was the α,β-unsaturated C-8 aldehyde moiety which, according to Taniguchi *et al*., was essential for activity [10]. However, this appears to be a contradiction with their own previous report in which **2** was devoid of antifungal activity, although it possess the α,β-unsaturated C-8 aldehyde moiety [7,10]. Fujita and Kubo also reported that **1** lost its antifungal activity against *Saccharomyces cerevisiae* in certain media, because of formation of a pyrrole between its aldehydes and the amines present in the medium [7]. Considering that, with the formation of a pyrrole, both aldehydes disappear, this report opens a new question: if one or both aldehydes are necessary for the antifungal activity.

In order to clarify the main features required for drimanes to display antifungal activities, we tested herein a series of 17 compounds **1**–**17** (most of them not previously assayed for antifungal activities) for antifungal properties against a unique panel of nine fungal strains, with a standardized methodology, in the same laboratory. To guarantee confident and reproducible results, the whole series was tested following the Clinical and Laboratory Standards Institute (CLSI) [11,12] standardized procedures. CLSI has established consensus procedures to facilitate the agreement among laboratories in measuring the susceptibility of yeasts (updated in 2008 as M-27 A3) and of filamentous fungi (updated in 2008 as M-38 A2) to antifungal agents, with broth dilution methods. The standardized parameters detailed in both documents include protocols for the preparation of antifungal stock solutions, dilutions for testing, inocula, culture medium, temperature and incubation time, endpoint definitions and reference MIC ranges for microdilution testing of both, the established and newly introduced antifungal agents.

Among the tested analogues of **1**, compounds **2**–**4** were isolated from *P. acuminatum* leaves and the remaining compounds **5**–**17** were prepared by isomerization, reduction, oxidation and methylenation reactions using the natural compounds **1**, **2** and **3** as starting materials. They were tested against three yeasts: *Candida albicans*, *S. cerevisiae* and *Cryptococcus neoformans*; three *Aspergillus* spp. (*A. flavus*, *A. fumigatus* and *A. niger*) and three dermatophytes: *Microsporum gypseum, Trichophyton rubrum * and *Trichophyton mentagrophytes* by using two end-points MIC_100_ and MIC_50_. A SAR analysis of the whole series, supported by conformational and electronic studies on some selected compounds is presented herein, with the aim of determining the stereo-electronic characteristics related to the antifungal behavior and thus contribute to the understanding of the structural properties of antifungal drimanes.

## 2. Results and Discussion

### 2.1. Chemistry

Sesquiterpenes **1**–**4** were obtained from *P. acuminatum* leaves as described previously [2]. Compounds **1**–**3** were used as starting materials for the preparation of compounds **5**–**17**.

#### 2.1.1. Compounds Obtained from **1**

**Scheme 1:**
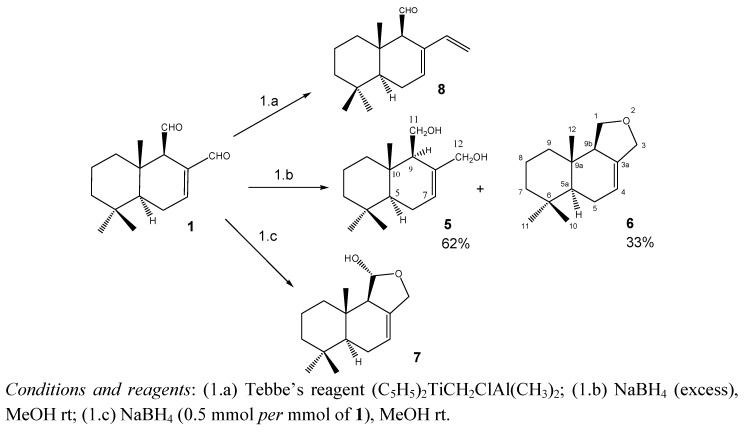
Modifications performed on **1** to obtain compounds **5**–**8**.

Scheme 1 shows the modifications performed on compound **1** that led to compounds **5**–**8**. Reduction of **1** with excess of NaBH_4_ in MeOH at rt produced (−)-(5*S*,10*S*)-(9*R*)-7-drimene-11,12-diol (**5**) and (−)-(5a*S*,9a*S*,9b*R*)-6,6,9a-trimethyl-1,3,5,5a,6,7,8,9,9a,9b-decahydronaphtho[1,2-c]furan (**6**). Isodrimeninol (**7**) was obtained by reduction of **1** with 0.5 mmol of NaBH_4_ for each mmol of drimane and compound **8** was a methylenation product obtained by treating **1** with one equivalent of the bulky Tebbe´s reagent, following previously reported procedures [13]. The identities of **5**, **7** and **8** were established by comparison with previously reported data [13,14,15,16]. Compound **6**, which was only previously reported once [16], is described in this work.

#### 2.1.2. Compounds Obtained from **2**

**Scheme 2:**
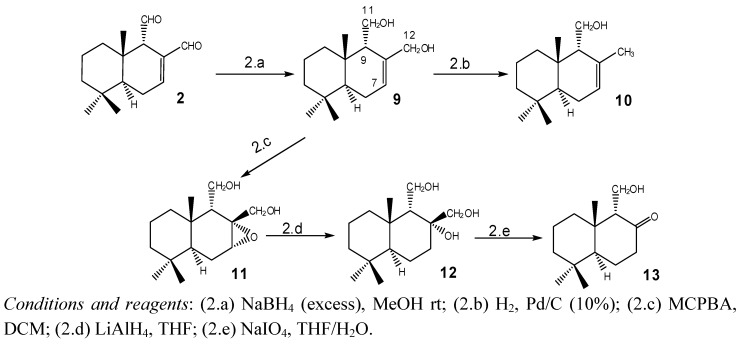
Modifications performed on **2** to obtain compounds **9**–**13**.

Scheme 2 shows the modifications performed on compound **2**. Reduction of **2** with excess NaBH_4_ in MeOH at r.t. produced (+)-(5S,10*S*)-(9*S*)-7-drimene-11,12 diol (isodrimendiol, **9**) [17] which, by catalytic hydrogenolysis using Pd/C (10% Pd), was converted into isodrimenol **10** [15,18]. Epoxidation of **9** with *m*-chloroperbenzoic acid (MCPBA) produced a single epoxide **11** in 75% yield. The 7-H signal in the ^1^H-NMR spectrum (δ 3.3, brdd, *J* = 5.2 Hz, 5.2 Hz) [19] and the signal for C-5, appearing at a δ 15.2 ppm lower than that corresponding to **9** in the ^13^C-NMR spectrum, confirmed the α configuration of **11**, as reported for similar compounds [20]. The epoxide **11** was refluxed with LiAlH_4_ in THF to afford the triol **12** in 85% yield. Oxidative degradation of **12** with sodium periodate gave the ketol **13** in 75% yield [21].

#### 2.1.3. Compounds Obtained from **3**

**Scheme 3:**
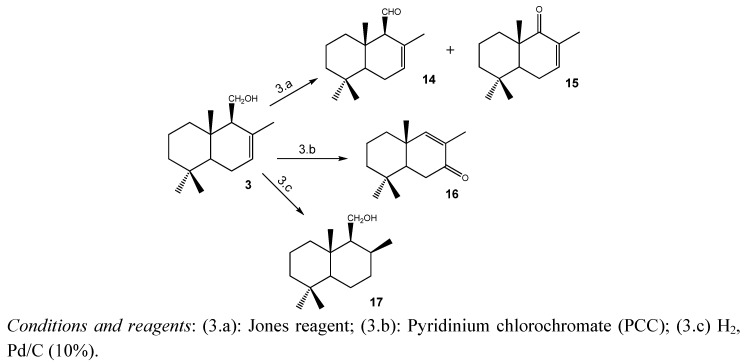
Modifications performed on **3** to obtain compounds **14**–**17**.

Scheme 3 shows the modifications performed on **3**. Oxidation of **3** with Jones reagent afforded drimenal **14** and the unsaturated ketone **15**. On the other hand, the unsaturated ketone **16** was obtained by treating **3** with the oxidation reagent pyridinium chlorochromate (PCC) [14]. Their structures were determined by comparison with the previously reported data [14,22,23]. In addition, catalytic hydrogenation of **3** using Pd/C (10% Pd), led to the saturated drimanol **17** whose spectroscopic data were in accordance to the literature [24].

### 2.2. Antifungal Activity

To carry out the antifungal evaluation, concentrations of naturally occurring drimanes **1**–**4** and semi-synthetic derivatives **5**–**17** up to 250 µg/mL were incorporated to growth media according to CLSI guidelines [11]. Table 1 summarizes the concentration of drimanes that completely inhibited (MIC_100_) the growth of nine opportunistic pathogenic fungi including yeasts (*C. albicans*, *C. neoformans*, and *S. cerevisiae*), hialohyphomycetes (*Aspergillus spp*.) as well as dermatophytes (*Microsporum* and *Trichophyton spp*). In addition to MIC determinations, the Minimum Fungicidal Concentration (MFC) of each active compound against this panel was accomplished by sub-culturing a sample of media from MIC tubes showing no growth, onto drug free agar plates (Table 1).

**Table 1:** Minimum Inhibitory Concentration (MIC_100_) and Minimum Fungicidal Concentration (MFC) of naturally occurring (**1**–**4**) and semi-synthetic (**5**–**17**) drimanes, against human opportunistic pathogenic fungi.

**Compounds**			*Yeasts*				*Aspergillus spp*			*Dermatophytes*		
Nº	Structure		*Ca*	*Sc*	*Cn*	*Afum*		*Afl*	*An*	*Mg*	*Tr*	*Tm*
**1**	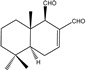	MIC_100_	3.9	15.6	7.8	I		I	I	62.5	62.5	62.5
		MFC	7.8	31.2	7.8	-		-	-	125	125	125
**2**	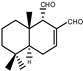											
		MIC_100_	62.5	62.5	62.5	I		I	I	62.5	62.5	62.5
		MFC	125	125	125	-		-	-	125	125	125
**3**	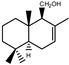											
		MIC_100_	I	I	125	I		I	I	62.5	62.5	62.5
		MFC	-	-	I	-		-	-	125	125	125
**4**	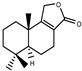											
		MIC_100_	I	250	I	I		I	I	125	62.5	62.5
		MFC	-	-	-	-		-	-	250	125	125
**5**	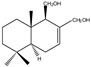											
		MIC_100_	125	250	250	I		I	I	250	250	250
		MFC	-	-	-	-		-	-	250	250	250
**6**	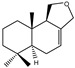											
		MIC_100_	250	250	125	I		I	I	62.5	62.5	62.5
		MFC	I	I	250	-		-	-	125	125	125
**7**	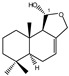											
		MIC_100_	250	250	125	I		I	I	31.2	31.2	31.2
		MFC	I	I	250	-		-	-	125	125	125
**8**	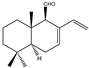											
		MIC_100_	250	250	125	I		I	I	62.5	62.5	62.5
		MFC	I	I	250	-		-	-	125	125	125
**9**	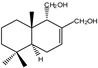											
		MIC_100_	250	250	125	I		I	I	125	125	250
		MFC	I	I	250	-		-	-	125	250	250
**10**	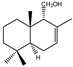											
		MIC_100_	125	125	31.2	I		I	I	125	62.5	250
		MFC	250	125	62.5	-		-	-	250	125	250
**11**	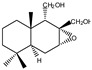											
		MIC_100_	I	I	I	I		I	I	I	I	I
		MFC	-	-	-	-		-	-	-	-	-
**12**	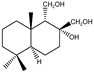	MIC_100_	I	I	I	I		I	I	I	I	I
		MFC	-	-	-	-		-	-	-	-	-
**13**	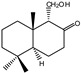	MIC_100_	I	I	I	I		I	I	250	250	250
		MFC	-	-	-	-		-	-	I	250	250
**14**	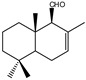	MIC_100_	125	125	31.2	I		I	I	62.5	62.5	125
		MFC	125	125	31.2	-		-	-	125	125	250
**15**	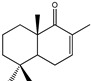	MIC_100_	I	I	250	I		I	I	250	250	250
		MFC	-	-	-	-		-	-	250	250	250
**16**	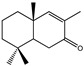	MIC_100_	250	250	250	I		I	I	250	250	250
		MFC	I	I	250	-		-	-	250	250	250
**17**	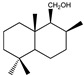	MIC_100_	I	I	I	I		I	I	I	I	I
		MFC	-	-	-	-		-	-	-	-	-
	St drugs Amph B Terbinafine Ketoconazole											
		MIC	0.78	0.50	0.25	0.50		0.50	0.50	0.12	0.075	0.075
		MIC	1.56	3.12	0.39	0.78		0.78	1.56	0.04	0.01	0.025
		MIC	0.5	0.5	0.25	0.12		0.5	0.25	0.05	0.025	0.025

*Ca*: *Candida albicans* ATCC 10231, *Sc*: *Saccharomyces cerevisiae* ATCC 9763, *C.*: *Cryptococcus* MIC_50_ (µg/mL) *neoformans* ATCC 32264; *An*: *Aspergillus niger* ATCC 9029, *Afu*: *Aspergillus fumigatus* ATCC 26934; *Afl*: *Aspergillus flavus* ATCC 9170, *Mg*: *Microsporum gypseum* C 115; *Tr*: *Trichophyton rubrum* C113, *Tm*: *Trichophyton mentagrophytes* ATCC 9972; Amph B: Amphotericin B. I: inactive (MIC > 250 µg/mL).

Regarding the sensitivity of the different fungi to drimanes, it can be stated that: (i) all compounds, but not **11**, **12** and **17**, exhibited moderate activity (MICs ≤ 250 µg/mL) against dermatophytes and yeasts; (ii) no member of the series was active against *Aspergillus* spp; (iii) all active compounds showed fungicidal rather than fungistatic properties against most fungi, since they not only inhibit but also kill them at Minimum Fungicidal Concentrations (MFC) ≤ 250 µg/mL.

### 2.3. Structure-Activity Relationships (SAR)

Since no differences in MICs among the members were observed against dermatophytes (one-dilution-step MIC changes are within the range of experimental error) [25], the SAR studies were performed on the activity of the series against yeasts (*C. albicans*, *S. cerevisiae* and *C. neoformans*) which showed striking differences.

For a better analysis of the differential behavior of these compounds against yeasts, we selected the clinically important fungi *C. neoformans* and *C. albicans* as targets. *C. neoformans* is a fungus thatremains an important life-threatening complication for immunocompromised hosts, particularly for patients who have undergone transplantation of solid organs [26]. Therefore, the activity of compounds against this fungus has a true clinical relevance. In turn, *C. albicans* is one of the leading causes of nosocomial blood stream infections worldwide [27].

Thus, compounds **1**–**10**, **14**–**16** that showed activity against at least one yeast were re-tested against the standardized strains *C. albicans* ATCC 10231 and *C. neoformans* ATCC 32264 determining, this time, MIC_50_ (the minimum concentration of compounds that inhibit 50% of growth), a less stringent end-point highly used [27] and recommended by CLSI [10]. MIC_50_ consistently represent the *in vitro* activity of compounds and many times provide a better correlation with other measurements of antifungal activity [27]. Results are shown in Table 2.

**Table 2:** MIC_50_ values of representative members of the drimane series against *C. albicans* ATCC 10231 (*Ca*) and *C. neoformans* ATCC 32264 (*Cn*).

MIC_50_ (µg/mL)			
	*Ca*	*Cn*	*LogP*
1	1.9	1.9	2.33
2	31.2	31.2	2.33
3	62.5	31.2	3.83
4	62.5	62.5	3.35
5	250	250	2.76
6	62.5	31.2	3.40
7	62.5	31.2	3.24
8	31.2	31.2	3.67
9	250	31.2	2.77
10	62.5	15.6	3.83
14	62.5	62.5	3.53
15	250	I	3.84
16	31.2	62.5	3.37
17	I	I	4.30
Amphotericin B	0.78	0.25	

From the analysis of the structures and the activities displayed by all compounds against *C. albicans* and *C. neoformans*, some structure-activity relationships can be extracted. They were grouped according to the influence of each structural feature such as Δ^7,8^-double bond in the drimane skeleton, configuration of C-9, presence of an aldehyde at C-9 (or at C-8) and presence of an α,β-unsaturated aldehyde moiety at C-8.

#### 2.3.1. Role of the Δ^7,8^-Double Bond in the Drimane Skeleton Alone and in α,β Position Respective to the Aldehyde on C-8

The Δ^7,8^**-**double bond in the drimane scheleton appears to be one of the necessary structural features for antifungal activity since inactive compounds **11**, **12**, **13** and **17** all lack this Δ**^7,8^-**double bond. The comparison of the activities of **3** with its saturated derivative **17** (devoid of antifungal activity), clearly reinforces this observation. A computer-assisted analysis supporting this observation is presented below, in Section 2.4.

In addition, it can be observed that the Δ**^7,8^-**double bond in active structures can be found either alone as in compounds **3**, **5**–**10** and **14**, or as being part of the **C**-8 α,β-unsaturated aldehyde moiety (constituting a Michael acceptor group) as in compounds **1** and **2**. The fact that both drimane structural types do display antifungal activity suggests that antifungal drimanes could not act *via* a Michael addition. This finding differs from the antifungal structural requirements of some other sesquiterpenes such as the sesquiterpenelactones (+)-costunolide and (−)-dehydrocostunolide isolated from *Centaurea* plants [28] and the synthetic tuberiferin and dehydrobrachylaenolide [29], whose antifungal activities were reported to be enhanced by a Michael acceptor group. Nevertheless, our findings are not in any way strange since many other diverse structural compounds not containing a Michael acceptor group, were found to display strong antifungal properties [30,31].

#### 2.3.2. Role of the Configuration of C-9

The comparison of activities of **1** and **2** shows that, in contrast to Fujita and Kubo’s previous report [7], both compounds do possess antifungal activity. However, the 9α-derivative **2** displays 16 and eight times lower activity than its epimer **1**, against *C. albicans* and *C. neoformans*, respectively. This result is consistent with Anke and Sterner’s [8] and Derita *et al*.’s [2] previous studies, who both found that **2** did display antifungal activities, but lower than **1**. The different antifungal activities displayed by compounds **1** and **2** are supported by their different electronic distributions which are presented in Section 2.4.

#### 2.3.3. Role of an Aldehyde at C-9 and of a CH_2_OH at C-8

The presence of an aldehyde group at C-9 appears not to be indispensable for activity, as can be observed in compounds **3**–**7**, **9**, **10**, **15** and **16**. Otherwise, the comparison of diol **9** with the monoalcohol **10**, suggests that a CH_2_OH at C-8 does not contribute to the activity either.

### 2.4. Conformational and Electronic Studies

To gain a better understanding of the above experimental results, computer-assisted conformational and electronic studies were performed on all compounds of the series. The purpose was to obtain precise information about the antifungal drimanes’ stereoelectronic characteristics that give support to the relationships between structure and activity.

#### 2.4.1. Conformational Analysis

**Figure 2:**
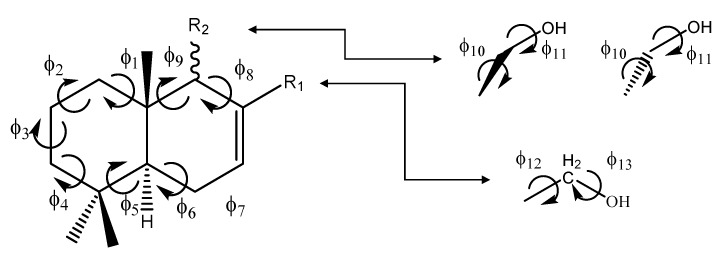
General representation of drimanes reported here, showing the torsional angles that determine the spatial ordering of rings A and B.

For the conformational analysis, each ring of the drimane scheleton was named A and B (Figure 2). The essential conformational problem of drimane sesquiterpenes, involves two aspects:

(a) The conformational behavior of torsional angles ф_1_-ф_5_ which determines the spatial ordering of ring A.
(b) The orientation of torsional angles ф_6_-ф_9_ which determines the overall shape of ring B. In this ring, they are also important torsional angles ф_10_ and ф_11_, which are related to CH_2_OH substituents as R_2_ or R_1_.

Our B3LYP/6311G(d.p) calculations indicated that a chair, and not a boat form, is the energetically preferred spatial ordering for ring A in compound **1**. The energy gap obtained between both conformers is 14.7 Kcal/mol, indicating that this compound is fairly rigid and possesses a restricted conformational flexibility. Similar results were obtained for all compounds possessing this structural feature like compounds **1**–**3**, **5**, **8**–**10** and **14**. Considering that, in these compounds, ring B is a cyclohexene, each of the three possible conformations: sofa (also called half-boat), half-chair and boat, were evaluated.

**Figure 3:**
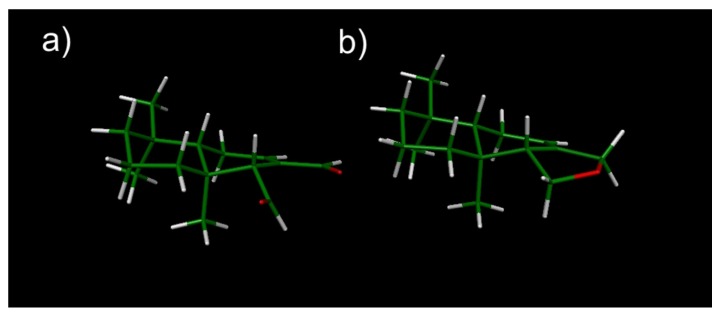
Spatial view of the lowest energy conformations obtained for compounds **1** (**a**) and **6** (**b**) from DFT (B3LYP/6-311G(d,p) optimizations. The chair and half-boat forms adopted by rings A and B respectively, may be appreciated in this figure.

Our theoretical results indicated that the so-called “half-boat” conformation is the highly preferred low-energy form Figure 3a which is in agreement with experimental data obtained for structurally related compounds [32,33,34]. Considering that all these compounds adopt similar low flexible conformations, it is clear that this would not be the cause of the different activities displayed against fungi. The same can be said for compounds **4**, **6** and **7**, for which our theoretical calculations indicate that the conformational flexibility is even more restricted than the observed for the above compounds as a consequence of the presence of a third fused ring. The energetically preferred conformation obtained for compound **6** is shown in Figure 3b. Closely similar conformations were obtained for compounds **4** and **7**.

Thus, the knowledge of the stereo-electronic properties of these drimanes will surely help to elucidate the structural requirements for the antifungal activity. For this analysis, Molecular Electrostatic Potentials (MEPs) are particularly valuable, because they allow the visualization and assessment of the capacity of a molecule to interact electrostatically with a putative-binding site [35,36,37].

**Figure 4:**
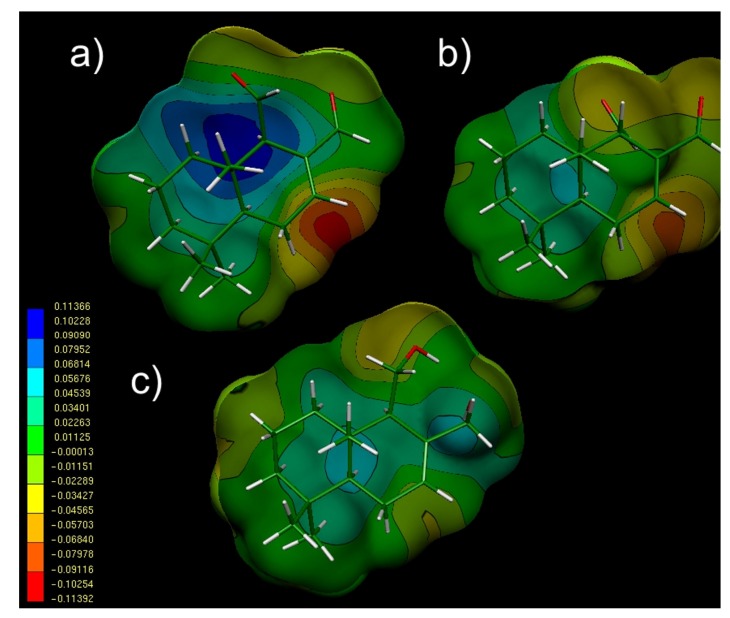
Electrostatic potential-encoded electron density surfaces of compounds **1** (**a**), **2** (**b**) and **5** (**c**). The surfaces were generated using B3LYP/6-311G++(d,p) single point calculations. The coloring represents electrostatic potential with red and blue indicating the electronegative or the electropositive areas respectively. The color-coded is shown at the left, where red is representing the most negative and the blue, the most positive values, respectively. Intermediate negative and positive values are represented by colours that downgrades from orange to light blue.

Figure 4 gives the MEPs obtained for compounds **1**, **2** and **5**, which showed high, moderate and low activities respectively. The MEP obtained for **1** (Figure 4a) exhibits three characteristic regions: a clear negative minimum value (deep red zone with V(r)_min_ ≈ −0.1139 el/au^3^) in the vicinity of Δ^7,8^ double bond; a positive region (deep blue zone with V(r)_max_ ≈ 0.1136 el/au^3^) located near the CH_3_ group at C_10_; and an extended hydrophobic zone (deep and light green zone with an almost neutral potential V(r)_med_≈ −0.0011 el/au^3^) in the rest of the molecule. Considering that **1** is the most active of the series, this MEP should account for the characteristics of the electronic general pattern for all the molecules possessing antifungal effect. Interestingly, the MEP obtained for its epimer **2** (Figure 4b), showed a marked electronegative zone near the double bond Δ^7,8^ similar to the showed by **1** but a little smaller. In turn, the positive zone is not observed in **2** which is a clear difference with **1**. Regarding compound **5**, concomitantly with its lower activity, it showed a red negative zone but considerably smaller than that of **2** (Figure 4c).

**Figure 5:**
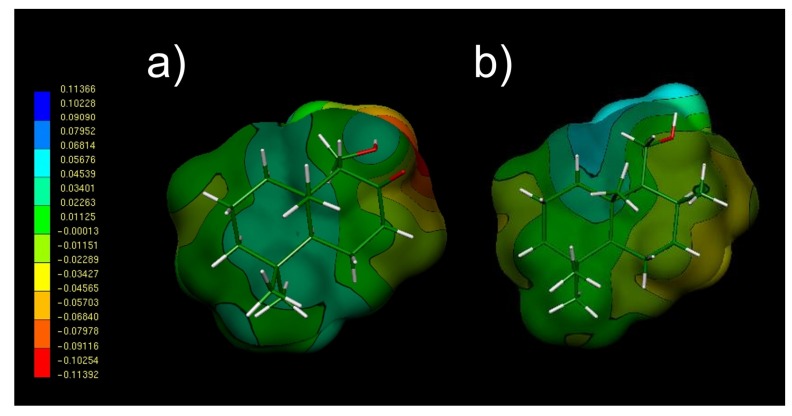
Molecular Electrostatic Potential (MEPs) obtained for compounds **13** (**a**) and **17** (**b**).

For the inactive compounds **13** and **17**, the electronegative zone is totally missing (Figure 5). This is an expected result considering that these compounds do not possess a double bond in such portion of their structures.

Finally, we calculated Log*P* (the logarithm of the partition coefficient in a biphasic system, e.g., n-octanol/water) for all drimanes which are shown in Table 2. It is known that Log*P* describes the macroscopic hydrophobicity of a molecule, which is a factor related to its ability to penetrate membranes of fungal cells and to reach the interacting sites, thus influencing the antifungal activity of compounds [38]. *LogP* values for the different active drimanes of the series varied from 2.33 to 3.84, which fall within the range of values observed for other antifungal compounds [31,39,40]. However, although these values indicate that they all are lipophilic compounds [39,41], Log*P* have similar values for drimanes with clear different activity, suggesting that this parameter would not have a direct correlation with the antifungal activity of the compounds of Table 2 when acting against *C. albicans* and *C. neoformans*.

Thus, the most active drimane **1** and its epimer **2**, both possess the same Log*P* value = 2.33 yet their activity is quite different. The same can be observed for compound **14** which has a Log*P* = 3.53 and displays lower activities. This behavior of drimanes was dissimilar to that of sesquiterpene lactones, for which a clear inverse relationship between polarity and antifungal activity was observed [28,29]. However, we must take into account that the sesquiterpene lactone skeletons are quite different from those of the drimane sesquiterpenes. In addition, different fungal species were used as the test species in each work; here, the yeasts *C. albicans* and *C. neoformans* were used, while in the previous reports the filamentous fungus *Cunninghamella echinulata* [28] and *Phycomyces blakesleeanus* were used [29]. It has been often observed completely different behaviors for certain compounds towards different type of fungi [42]. As the authors stated in [28], there is not much information about the chemical structure-antifungal activity relationships for sesquiterpenoids and deeper studies should be necessary. In this work, we add information that could be useful for a better knowledge of drimane sesquiterpenes behavior against human opportunistic and pathogenic fungi.

## 3. Experimental

### 3.1. Chemistry

Melting points were determined in a Büchi melting point apparatus and are uncorrected. The ^1^H- and ^13^C-NMR spectra were run on a Bruker DPX 300 spectrometer operating at 300 and 75 MHz, respectively, using chloroform-*d* as solvent and tetramethylsilane as internal standard. The mass spectra were scanned on a Hewlett Packard HP Engine-5989 spectrometer (equipped with a direct inlet probe) operating at 70 eV.

#### 3.1.1. Isolation of Natural Compounds **1**–**4**

Compounds **1**–**4** were isolated from DCM extracts of leaves of *P. acuminatum* Kunth. (Polygonaceae) collected on the bank of Coronda River at Puerto Gaboto, Santa Fe Province, Argentina, in March 2010. The plant was identified by Prof. Susana Gattuso and a voucher specimenwas deposited at the Herbarium of the Vegetal Biology Area of UNR, Suipacha 531, Rosario, Argentina (UNR # 2359). The extraction methodology and isolation of pure compounds was performed according to reported procedures [1]. Compounds **1**–**4** were identified by micromelting point, optical rotation and spectroscopic data, including ^1^H- and ^13^C-NMR [43,44,45,46,47] and were compared with authentic samples obtained previously in our laboratory [1,48,49] for polygodial (**1**) and isopolygodial (**2**) or with literature data [50,51], for drimenol (**3**) and for confertifolin (**4**) [44,52].

#### 3.1.2. General Procedure for Synthesis of **5** and **6**

To a magnetically well-stirred solution of **1** (2.1 mmol) in MeOH (25 mL) acidified with 5 drops of 1 M HCl, NaBH_4_ (8.4 mmol) was added carefully in small portions. The mixture was refluxed at 50 °C for 4 h and most of the solvent was evaporated *in vacuo*. Water (20 mL) was added cautiously and extractions with EtOAc were performed. The organic phase was dried with MgSO_4_, evaporated and submitted to column chromatography (CC) to give compounds **5** (1.3 mmol) and **6** (0.7 mmol) in yields of 62% and 33%, respectively.

*(*−*)-(5S,10S)-(9R)-7-Drimene-11,12diol (drimendiol)* (**5**): White solid; mp: 74 °C; [α]_D_^25^ −8° (CHCl_3_, *c.* = 1.0). IR (film) ν_max_ 3286, 2924 cm^−1^. ^1^H-NMR: δ 5.78 (1H, *m*, H-7); 4.33 (1H, *d*, *J* = 11.9 Hz, H-12B); 4.00–3.90 (4H, *m*, H-11B, 12A and 2OH); 3.64 (1H, *dd*, *J* = 8.7 and 10.4 Hz, H-11A); 2.15–1.90 (4H, *m*, H-1β, 6α, 6β and 9); 1.58–1.40 (3H, *m*, H-2α, 2β and 3β); 1.24–1.10 (3H, *m*, H-1α, 3α and 5); 0.88 and 0.86 (6H, 2*s*, 13-Me and 14-Me); 0.75 (3H, *s*, 15-Me). ^13^C-NMR: δ 136.9 (C-8); 127.1 (C-7); 67.3 (C-12); 61.2 (C-11); 54.4 (C-5); 49.4 (C-9); 42.0 (C-3); 39.3 (C-1); 35.6 (C-10); 33.2 (C-13); 32.9 (C-4); 23.5 (C-6); 21.9 (C-14); 18.8 (C-2); 14.5 (C-15). MS *m/z* (EI): 238 [M^+^]. All these data are coincident with the literature [13,16].

*(*−*)- (5aS,9aS,9bR)-6,6,9a-Trimethyl-1,3,5,5a,6,7,8,9,9a,9b-decahydronaphtho[1,2-c]furan* (**6**): [α]_D_^25^ −17° (CHCl_3_, *c.* = 1.0), IR (film) ν_max_ 2980, 1660, 1460, 1380, 1365 cm^−1^. ^1^H-NMR: δ 5.80 (1H, *m*, H-4); 4.35 (1H, *dd*, *J* = 1.1 and 12.0 Hz, H-3β); 3.99 (1H, *d*, *J* = 12.0 Hz, H-3α); 3.91 (1H, *dd*, *J* = 2.1 and 10.9 Hz, H-1α); 3.68 (1H, *dd*, *J* = 8.3, 10.9 Hz, H-1β); 2.15 (1H, *m*, H-9b); 2.20–1.20 (9H, *m*, H-9α and β, H-8α and β, H-7α and β, H-5α and β, H-5a); 0.90 and 0.88 (6H, 2*s*, 12-Me and 11); 0.77 (3H, *s*, 10-Me). ^13^C-NMR: δ 136.9 (C-3a), 127.4 (C-4); 67.4 (C-3); 61.4 (C-1); 54.4 (C-9b); 49.4 (C-5a); 42.0 (C-7); 39.3 (C-9); 35.6 (C-9a); 33.2 (C-11); 32.9 (C-6); 23.6 (C-5); 21.9 (C-10); 18.8 (C-8); 14.5 (C-12). MS *m/z* (EI): 220 [M^+^]. This is the first time that this compound is described in detail.

#### 3.1.3. Synthesis of (−)-(1*R*,5a*S*,9a*S*,9b*R*)-6,6,9a-Trimethyl-1,3,5,5a,6,7,8,9,9a,9b-decahydro naphto[2,1c]furan-1-ol (isodrimeninol, 7)

Compound **1** (0.55 mmol) was dissolved in MeOH (10 mL) acidified with 3 drops of 1 M HCl and NaBH_4_ (0.27 mmol) was added carefully in small portions. The mixture was stirred during 30 min at 0 °C and then for 3 h at rt. Water (10 mL) was added cautiously and extractions with EtOAc were performed. The organic phase was dried with MgSO_4_, evaporated and submitted to CC to give compound **7** (0.39 mmol) in 71% yield. Colorless oil; [α]_D_^25^ −12° (CHCl_3_, *c.* = 1.0); IR (film) ν_max_ 3400, 1630, 1390, 1370, cm^−1^. ^1^H-NMR: δ 5.52 (1H, *m*, H-4); 5.29 (1H, br*d*, *J =* 2.2 Hz, H-1); 4.50 (1H, br*d*, *J =* 11.3 Hz, H-3β); 4.19 (1H, br*d*, *J =* 11.3 Hz, H-3α); 3.04 (1H, br*s*, -OH); 2.21 (1H, br*d*, *J =* 2.2, H-9b); 2.13 (1H, *m*, H-5α); 1.92 (1H, *m*, H-5β); 1.80 (1H, *ddd*, *J =* 2.7, 4.7 and 13.2 Hz, H-9β); 1.61 (1H, *m*, H-8β); 1.49 (2H, *m*, H-8α and 7β); 1.31 (3H, *m*, H-5a, 9α and 7α); 0.93; 0.90 (6H, 2*s*, 10-Me and 11); 0.83 (3H, *s*, 12-Me). ^13^C-NMR: δ 136.5 (C-3a), 117.1 (C-4), 99.4 (C-1), 68.9 (C-3), 61.6 (C-9b), 49.8 (C-5a), 42.4 (C-7), 39.8 (C-9), 33.4 (C-9a), 33.1 (C-11), 32.9 (C-6), 23.6 (C-5), 21.5 (C-10), 18.5 (C-8), 14.0 (C-12). MS *m/z* (EI): 236 [M^+^]. All these data agree with the literature values [14].

#### 3.1.4. Synthesis of (−)-(1*R*,4a*S*,8a*S*)-5,5,8a-Trimethyl-2-vinyl-1,4,4a,5,6,7,8,8a-octahydro naphtalene-1-carbaldehyde (**8**) [11]

Compound **1** (2.14 mmol) was dissolved in anhydrous THF (5 mL), the solution was stirred at 0 °C under nitrogen, and Tebbe´s reagent [(C_5_H_5_)_2_TiCH_2_ClAl(CH_3_)_2_] (4 mL of a 0.5M solution in toluene, 2.0 mmol) was added dropwise. The dark brown solution was allowed to warm to room temperature for 10 min, 0.1 M NaOH solution was added (10 drops) and the slurry was filtered through a short pad of silica gel. The filtrate was evaporated and purified by CC to give compound **8** as a colorless oil, yield 25%; [α]_D_^25^ −17° (CHCl_3_, *c.* = 1.0); IR (film) ν_max_ 3090, 2720, 1719, 1643, 1607 cm^−1^. ^1^H-NMR: δ 9.48 (1H, *d*, *J =* 5.1 Hz, CHO); 6.35 (1H, *dd*, *J =* 11.2 and 17.9 Hz, Hc); 6.14–6.11 (1H, *m*, H-3); 4.93 (1H, *d*, *J =* 11.2 Hz, Ha); 4.82 (1H, *d*, *J =* 17.9 Hz, Hb); 2.82 (1H, br*s*, H-1); 2.29–2.19 (2H, *m*, H-4α and β); 1.87–1.81 (1H, *m*, H-8β); 1.54–1.15 (6H, *m*, H-8α, H-7α and β, H-6α and β, H-4a); 1.03 (3H, *s*, 8a-Me); 0.95 and 0.91 (6H, 2*s*, 5-Meα and β). ^13^C-NMR: δ 206.7 (CHO); 138.5 (=CH); 132.8 (C-3); 131.5 (C-2); 112.4 (=CH_2_); 62.6 (C-1); 48.9 (C-4a); 41.9 (C-6); 40.2 (C-8); 37.3 (C-8a); 33.3 (5-Me); 33.1 (C-5); 24.1 (C-4); 22.3 (5-Me); 18.1 (C-7); 15.5 (8a-Me). MS *m/z* (EI): 232 [M^+^]. All these data are coincident with the literature [13].

#### 3.1.5. Synthesis of (+)-(5*S*,10*S*)-(9*S*)-7-Drimene-11,12diol (isodrimendiol, 9)

To a magnetically well-stirred solution of **2** (1.06 mmol) in MeOH (20 mL) acidified with 5 drops of 1 M HCl, NaBH_4_ (4.2 mmol) was added carefully in small portions. The mixture was refluxed at 50 °C for 4 h and most of the solvent was evaporated *in vacuo*. Water (20 mL) was added cautiously and extractions with EtOAc were performed. The organic phase was dried with MgSO_4_, evaporated and submitted to CC to give compound **9** (0.83 mmol) as white solid, yield 78%, mp: 129 °C; [α]_D_^25^+10° (CHCl_3_, *c.* = 1.0). IR (film) ν_max_ 3286, 2924, 1640, 1045 cm^−1^. ^1^H-NMR: δ 5.83 (1H, *t*, *J =* 3.5 Hz, H-7); 4.13 (1H, *d*, *J =* 12.0 Hz, H-12B); 4.00 (1H, *d*, *J =* 12.0 Hz, H-12A); 3.92 (1H, *dd*, *J =* 4.5 and 11.0 Hz, H-11B); 3.64 (1H, *dd*, *J =* 5.4 and 11.0 Hz, H-11A); 2.60 (2H, br*s*, 2OH); 2.20–1.90 (3H, *m*, H-6α and β, H-9β); 1.67–1.20 (7H, *m*, H-1α and β, H-2α and β, H-3α and β, H-5); 0.92 (6H, 2*s*, 15-Me and 14-Me); 0.88 (3H, *s*, 13-Me). ^13^C-NMR: δ 137.2 (C-8); 127.5 (C-7); 67.7 (C-12); 63.1 (C-11); 54.1 (C-5); 43.4 (C-9); 42.7 (C-3); 36.5 (C-1); 35.9 (C-10); 33.1 (C-4); 33.0 (C-15); 24.3 (C-6); 22.0 (C-14); 21.7 (C-13); 18.8 (C-2). MS *m/z* (EI): 238 [M^+^]. All these data are in agreement with the literature values [16].

#### 3.1.6. Synthesis of (+) [(1*R*,4a*S*,8a*S*)-2,5,5,8a-Tetramethyl-1,4,4a,5,6,7,8,8a-octahydro naphthalen-1-yl]methanol (isodrimenol, **10**)

Diol **9** (0.34 mmol) was dissolved in DCM (15 mL) and catalytic quantities of Pd/C (10% Pd) were added. The mixture was left under a hydrogen atmosphere during 4 h and the reaction product was filtered, concentrated and purified by CC. Compound **10** (0.29 mmol) was thus obtained in 85% yield; [α]_D_^25^ +10° (CHCl_3_, *c.* = 1.0); IR (film) ν_max_ 3405, 2922, 1620 cm^-1^; ^1^H-NMR: δ 5.57 (1H, *m*, H-7); 3.74 (2H, br*d*, *J =* 3.0, H-11A and B); 1.74 (3H, br*s*, 12-Me); 2.10–1.20 (10H, *m*, H-1α and β, H-2α and β, H-3α and β, H-5, H-6α and β, H-9); 0.93 (3H, *s*, 15-Me); 0.91 and 0.87 (6H, 2*s*, 13-Me and 14-Me). ^13^C-NMR: δ 131.1 (C-8); 124.6 (C-7); 61.3 (C-11); 57.6 (C-5); 43.4 (C-9); 42.7 (C-3); 36.8 (C-1); 36.1 (C-10); 33.2 (C-13); 33.0 (C-4); 24.0 (C-6); 23.0 (C-14); 22.2 (C-12); 21.7 (C-2); 18.8 (C-15). MS *m/z* (EI): 222 [M^+^]. All these data are in agreement with the literature [14,17].

#### 3.1.7. Synthesis of (−)-7*α*,8-Epoxy-(9*S*)-drimane-11,12-diol (**11**) [20]

To a solution of diol **9** (4.2 mmols) in CH_2_Cl_2_ (30 mL) MCPBA acid (5.4 mmol) was added in small portions at rt during 10 min. Stirring was continued at rt for 35 min. The reaction mixture was washed with NaHCO_3_ and water, dried and concentrated. The residue was chromatographed over silica gel to afford compound **11** (75% yield); mp: 105 °C; [α]_D_^25^ −69° (CHCl_3_, *c.* = 1.0); IR (film) ν_max_ 3280, 3120, 3050 cm^−1^. ^1^H-NMR: δ 3.95–3.45 (4H, *m*, H-11A and B, H-12A and B); 3.36 (1H, H-7); 2.1 (1H, *dd*, *J =* 4.5 and 15.3 Hz, H-9); 1.74–1.64 (2H, *m*, H-6α and β); 1.61–1.10 (7H, *m* H-1α and β, H-2α and β, H-3α and β, H-5); 0.96 (3H, *s*, 15-Me); 0.89 and 0.86 (6H, 2*s*, 13-Me and 14-Me). ^13^C-NMR: δ 66.6 (C-12); 62.0 (C-8); 60.6 (C-11); 56.8 (C-7); 48.3 (C-9); 42.4 (C-3); 39.0 (C-5); 36.2 (C-1); 34.9 (C-10); 32.9 (C-15); 32.8 (C-4); 22.8 (C-6); 22.7 (C-14); 22.2 (C-13); 18.3 (C-2). MS *m/z* (EI): 254 [M^+^]. All these data are in agreement with the literature [20].

#### 3.1.8. Synthesis of (−)-(9*S*)-Drimane-8,11,12-triol (**12**) [20]

To a stirred mixture of LiAlH_4_ (16 mmol) in dry THF (50 mL), a solution of the epoxide **11** (4.5 mmol) in dry THF (30 mL) was added and the mixture was heated at reflux temperature under nitrogen for 4 h. The excess of reagent was decomposed by the addition of EtOAc and an aqueous solution of HCl (10%). The mixture was extracted with EtOAc and the organic phase was washed with NaHCO_3_ and water, dried and concentrated to give compound **12** (85% yield); mp: 118 °C; [α]_D_^25^ −41° (CHCl_3_, *c.* = 1.0); IR (film) ν_max_ 3400, 3280, 3120 cm^−1^. ^1^H-NMR: δ 4.0 (2H, *m*, H-12A and B); 3.83–3.60 (3H, *m*, H-11A and B and 1OH); 3.33 (1H, br*s*, 1OH); 2.58 (1H, br*s*, 1OH); 1.89 (1H, *m*, H-9); 1.80–1.10 (11H, *m*, H-1α and β, H-2α and β, H-3α and β, H-5, H-6α and β, H-7α and β); 1.05 (3H, *s*, 15-Me); 0.87 and 0.80 (6H, 2*s*, 13-Me and 14-Me). ^13^C-NMR: δ 76.4 (C-8); 69.5 (C-12); 60.9 (C-11); 56.9 (C-5); 48.6 (C-9); 42.1 (C-3); 37.6 (C-10); 37.4 (C-1); 33.4 (C-4); 33.1 (C-7); 33.0 (C-15); 23.7 (C-14); 21.8 (C-13); 20.0 (C-6); 18.8 (C-2). MS *m/z* (EI): 256 [M^+^]. All these data are in agreement with the literature [20].

#### 3.1.9. Synthesis of (−)-11-Hydroxy-(9*R*)-12-nordriman-8-one (**13**) [20]

To a stirred solution of NaIO_4_ (3.53 mmol) in water (15 mL), a solution of the triol **12** (2.54 mmol) in MeOH (10 mL) was added at rt during 1.5 h. The reaction solution was extracted with EtOAc and the organic phase was washed with NaHCO_3_ and water, dried and concentrated to give compound **13** (90% yield); mp: 112 °C; [α]_D_^25^ −22° (CHCl_3_, *c.* = 1.0); IR (film) ν_max_ 3280, 1720 cm^−1^. ^1^H-NMR: δ 3.98 (1H, br*d*, *J =* 10 Hz, H-11A); 3.91 (1H, br*d*, *J =* 10 Hz, H-11B); 2.36–2.09 (2H, *m*, 2H-7); 2.37 (1H, *dd*, *J =* 4.3 and 1.8 Hz, H-9β); 2.11 (1H, *ddd*, *J =* 1.6, 6.2 and 9.8 Hz, H-7); 1.95 (1H, *m*, H-7); 1.70–1.16 (9H, *m*, H-1α and β, H-2α and β, H-3α and β, H-5, H-6α and β); 0.94 (3H, *s*, 15-Me); 0.92 and 0.84 (6H, 2*s*, 13-Me and 14-Me). ^13^C-NMR: δ 214.8 (C-8); 67.0 (C-9); 60.1 (C-11); 46.0 (C-5); 42.2 (C-3); 40.0 (C-10); 39.3 (C-1); 36.8 (C-7); 33.5 (C-15); 33.4 (C-4); 23.2 (C-6); 22.2 (C-13); 22.0 (C-14); 18.5 (C-2). All these data agree with the literature values [20].

#### 3.1.10. General Procedure for Synthesis of **14** and **15** [13,16]

A magnetically stirred and cold (0 °C) solution of the alcohol substrate **3** (10 mmol) in acetone (50 mL) was titrated with Jones reagent, until the orange-brown color of the reagent persisted for 30 seconds. The reaction mixture was stirred for an additional 1 h at rt, and then isopropanol (1 mL) was added dropwise to destroy any excess of reagent. Water (100 mL) was added, and the suspension was extracted with AcOEt, dried with MgSO_4_, concentrated, and purified by column chromatography to give compounds **14** (0.68 mmol) and **15** (0.85 mmol) with yields of 30 and 38%, respectively.

*(*−*)-(1S,8aS)-2,5,5,8a-Tetramethyl-1,4,4a,5,6,7,8,8a-octahydronaphthalene-1-carbaldehyde* (*drimenal*, **14**): colorless oil, [α]_D_^25^ −20° (CHCl_3_, *c.* = 1.0); IR (film) ν_max_ 2923, 2851, 1714 cm^−1^. ^1^H-NMR: δ 9.69 (1H, *d*, *J =* 5.1 Hz, H-11); 5.70 (1H, br*s*, H-7); 2.59 (1H, *m*, H-9); 2.05 (2H, *m*, H-6α and β); 1.70–1.10 (7H, *m*, H-1α and β, H-2α and β, H-3α and β, H-5); 1.62 (3H, *s*, 12-Me); 1.05 (3H, *s*, 15-Me); 0.90 and 0.85 (6H, 2*s*, 13-Me and 14-Me). ^13^C-NMR: δ 206.7 (C-11); 127.8 (C-8); 125.5 (C-7); 67.6 (C-9); 49.1 (C-5); 42.0 (C-3); 40.4 (C-1); 37.0 (C-10); 33.3 (C-14); 33.0 (C-4); 23.7 (C-6); 22.1 (C-13); 21.6 (C-12); 18.3 (C-2); 15.7 (C-15). MS *m/z* (EI): 220 [M^+^]. All these data are in agreement with the literature [16,21].

*(*−*)-(4aS,8aS)-2,5,5,8a-Tetramethyl-4a,5,6,7,8,8a-hexahydro-4H-naphtalen-1-one* (**15**): white solid; mp: 81 °C; [α]_D_^25^ −74° (CHCl_3_, *c.* = 1.0); IR (film) ν_max_ 2923, 1663 cm^−1^. ^1^H-NMR: δ 6.69–6.67 (1H, *m*, H-3); 2.30–2.25 (2H, *m*, H-4α and β); 1.89 (1H, br*d*, *J =* 13.4 Hz, H-8β); 1.75 (3H, *d*, *J =* 2.0 Hz, Me-2); 1.70–1.10 (6H, *m*, H-8α, H-7α and β, H-6α and β, H-4a); 1.04 (3H, *s*, Me-8a); 0.99 and 0.91 (6H, 2*s*, Me-5α and β). ^13^C-NMR: δ 205.9 (C-1); 143.4 (C-3); 132.9 (C-2); 49.4 (C-4a); 45.1 (C-8a); 41.6 (C-6); 33.6 (C-5); 33.2 (C-8); 32.3 (Me-5); 24.4 (C-4); 22.3 (Me-5); 18.2 (C-7); 17.1 (Me-8a); 16.4 (Me-2). MS *m/z* (EI): 206 [M^+^]. All these data are in agreement with the literature [13,16,22].

#### 3.1.11. Synthesis of (−)-(4a*S*,8a*S*)-3,4a,8,8-Tetramethyl-4a,5,6,7,8,8a-hexahydro-1*H*-naphtalen-2-one (**16**) [14,17]

To a magnetically stirred solution of the alcohol substrate **3** (10 mmol) in DCM (50 mL), pyridinium chlorochromate (10 mmol) was added. The brown suspension was stirred overnight, until thin layer chromatography analysis showed the disappearance of the starting material. Celite (500 mg) and EtOAc (50 mL) were added to the reaction mixture, and the slurry was filtered through a short pad of silica gel, washing copiously with EtOAc. The filtrate was dried, evaporated, and purified by column chromatography to give compound **16** (1.2 mmol) with 60% yield as a colorless oil; [α]_D_^25^ −7° (CHCl_3_, *c.* = 1.0); IR (film) ν_max_ 2956, 1704, 1673 cm^−1^. ^1^H-NMR: δ 6,39 (1H, *d*, *J =* 1.3 Hz, H-4); 2.49 (1H, *dd*, *J =* 3.9 and 17.4 Hz, H-1α); 2.34 (1H, *dd*, *J =* 14.0 and 17.4 Hz, H-1β); 1.72 (3H, *d*, *J =* 1.3 Hz, Me-3); 1.72 (1H, br*dd*, *J =* 13.9 and 3.9 Hz, H-5β); 1.70–1.10 (6H, *m*, H-5α, H-6α and β, H-7α and β, H-8a); 1.07 (3H, *s*, Me-4a); 0.91 (3H, *s*, Me-8); 0.88 (3H, *s*, Me-8). ^13^C-NMR: δ 201.6 (C-2); 158.3 (C-4); 131.2 (C-3); 50.6 (C-8a); 41.2 (C-7); 38.4 (C-5); 36.9 (C-4a); 35.4 (C-1); 32.8 (C-8); 32.2 (Me-8); 20.9 (Me-8); 18.6 (C-6); 18.5 (Me-4a); 15.5 (Me-3). MS *m/z* (EI): 206 [M^+^]. All these data are in agreement with the literature [13,16,22].

#### 3.1.12. Synthesis of (+) [(1*S*,2*S*,8a*S*)-2,5,5,8a-Tetramethyldecahydronaphthalen-1-yl]methanol (drimanol, **17**)

Drimenol (**3**, 0.36 mmol) was dissolved in DCM (10 mL) and catalytic quantities of Pd/C (10% Pd) were added. The mixture was left under a hydrogen atmosphere during 4 h and the reaction product was filtered, concentrated and purified by CC to obtain compound **17** (75% yield); [α]_D_^25^ +15° (CHCl_3_, *c.* = 1.0); IR (film) ν_max_ 3620, 2950, 1380 cm^−1^. ^1^H-NMR: δ 3.86 (1H, *dd*, *J =* 4.5 and 10.7 Hz, H-11B); 3.61 (1H, *dd*, *J =* 4.5 and 10.7 Hz, H-11A); 2.17 (1H, *m*, H-8); 1.65–1.10 (12H, *m*, H-1α and β, H-2α and β, H-3α and β, H-5, H-6α and β, H-7α and β, H-9); 0.98 (3H, *d*, *J =* 7.5 Hz, 12-Me); 0.88 and 0.83 (9H, 3*s*, 13-Me and 15-Me). ^13^C-NMR: δ 61.0 (C-11); 56.5 (C-5); 55.7 (C-9); 41.9 (C-3); 39.9 (C-1); 37.6 (C-10); 34.5 (C-7); 33.6 (C-14); 33.2 (C-4); 28.5 (C-8); 21.6 (C-13); 18.4 (C-6); 17.5 (C-2); 17.1 (C-15), 15.6 (C-12). MS *m/z* (EI): 224 [M^+^]. All these data are in agreement with the literature [23].

### 3.2. Antifungal Evaluation

#### 3.2.1. Microorganisms and Media

For the antifungal evaluation, standardized strains from the American Type Culture Collection (ATCC), Rockville, MD, USA, and CEREMIC (CCC), Centro de Referencia en Micología, Facultad de Ciencias Bioquímicas y Farmacéuticas, Suipacha 531 (2000)-Rosario, Argentina, were used in a first instance screening: *C. albicans* ATCC 10231, *S. cerevisiae* ATCC 9763, *C. neoformans* ATCC 32264, *A. flavus* ATCC 9170, *A. fumigatus* ATTC 26934, *A. niger* ATCC 9029, *T. rubrum* CCC 110, *T. mentagrophytes* ATCC 9972, and *M. gypseum* CCC 115.

Strains were grown on Sabouraud-chloramphenicol agar slants for 48 h at 30 °C, maintained on slopes of Sabouraud-dextrose agar (SDA, Oxoid), and sub-cultured every 15 days to prevent pleomorphic transformations. Inocula of cells or spore suspensions were obtained according to reported procedures and adjusted to 1–5 × 10^3^ cells/spores with colony forming units (CFU)/mL [11,12].

#### 3.2.2. Antifungal Susceptibility Testing

Minimum inhibitory concentration (MIC) of each compound was determined by using broth microdilution techniques according to the guidelines of the CLSI for yeasts (M27-A3) and for filamentous fungi (M 38 A2) [11,12]. MIC values were determined in RPMI-1640 (Sigma, St. Louis, MO, USA) buffered to pH 7.0 with MOPS. Microtiter trays were incubated at 35 °C for yeasts and hialohyphomycetes and at 28–30 °C for dermatophytes strains in a moist, dark chamber, and MICs were visually recorded at 48 h for yeasts, and at a time according to the control fungus growth, for the rest of fungi.

For the assay, stock solutions of pure compounds were two-fold diluted with RPMI from 250 to 0.98 µg/mL (final volume = 100 µL) and a final DMSO concentration ≤1%. A volume of 100 µL of inoculum suspension was added to each well with the exception of the sterility control where sterile water was added to the well instead. Ketoconazole, terbinafine and amphotericin B were used as positive controls.

Endpoints were defined as the lowest concentration of drug resulting in total inhibition (MIC_100_) of visual growth compared to the growth in the control wells containing no antifungal. MIC_50_ was defined as the lowest concentration of a compound that inhibited 50% of the growth control, respectively (culture media with the microorganism but without the addition of any compound), and was determined spectrophotometrically with the aid of a VERSA Max microplate reader (Molecular Devices, Sunnyvale, CA, USA).

The Minimum Fungicidal Concentration (MFC) of each compound against each strain was also determined as follows: After determining the MIC_100_, an aliquot of 5 µL sample was withdrawn from each clear well of the microtiter tray and plated onto a 150-mm RPMI-1640 agar plate buffered with MOPS (Remel, Lenexa, KS, USA.). Inoculated plates were incubated at 30 °C, and MFCs were recorded after 48 h. The MFC was defined as the lowest concentration of each compound that resulted in total inhibition of visible growth.

### 3.3. Calculations Methods

The DFT calculations, full geometry optimization and calculation of the harmonic vibrational frequencies were performed using the GAUSSIAN 03 program [53]. Density Functional Theory (DFT) calculations were employed in order to properly account for the electron correlation effects. The widely employed hybrid method denoted by B3LYP was used, along with the double-zeta split valence basis set 6-311G(d,p). This method includes a mixture of HF and DFT exchange terms and the gradient corrected correlation functional of Lee, Yang and Parr [54,55], as proposed and parameterized by Becke [56,57].

A preliminary conformational search was carried out using the GASCOS algorithm [58,59,60] combined with PM6 an RHF/3-21G calculations. All the conformations obtained using this calculations were confirmed from B3LYP/6311G(d, p) computations. With any conformational search, it is very important to examine the structures obtained to make sure that they are true minima and not transition structures or other structures with very low or zero forces on the atoms (stationary points). Thus, minima were characterized through harmonic frequency analysis employing B3LYP/6311G(d,p) calculations.

The electronic studies were carried out using molecular electrostatic potentials (MEPs) [61]. The low-energy conformations were obtained from our conformational search. Subsequently, single point calculations were carried out. Thus, these MEPs were calculated using B3LYP/6-311G++(d,p) wave functions and MEPs graphical presentations were created using the MOLEKEL program [62].

## 4. Conclusions

Results obtained here were aimed at contributing to clear up the main structural features necessary for the antifungal activity of drimanes (that have been the subject of contradictory statements in the literature) by testing them against a unique panel of fungal strains, with a standardized methodology, in the same laboratory.

Regarding the absolute configuration of C-9, here we found that compounds with an aldehyde in any of the two possible configurations did possess antifungal activity against *C. albicans* and *C. neoformans*, although the 9α-derivative **2** displays 16 and eight times lower activity than the 9β-one **1***.* This result is in contrast with Fujita and Kubo [7], but consistent with Anke and Sterner’s [8] and Derita *et al*.’s [2] previous studies, who both found that **2** did display antifungal activities but lower than those of compound **1**.

Another result demonstrated that aldehydes at C-9 or C-8 or a CH_2_OH at C-8 would not be a requisite for activity. In addition, this work showed that the Δ^7,8^**_-_**double bond in the drimane scheleton should be considered one of the necessary structural features for antifungal activity, either alone as in compounds **3**, **5**–**10** and **14**, or as part of a **C**-8 α,β-unsaturated aldehyde moiety (constituting a Michael acceptor group) as in compounds **1** and **2**. The fact that both drimane structural types do display antifungal activity clearly suggested that antifungal drimanes would not act *via* a Michael addition. This finding differs from Taniguchi’s report [10]. However, as explained in the Introduction, the statement of Taniguchi *et al*. in [10] contradicted their own previous report [7] in which **2** was shown to be devoid of antifungal activity, although it possessed the α,β-unsaturated C-8 aldehyde moiety. In addition, results obtained here showed that the electronic characteristics of drimanes play an important role in their antifungal behavior. Thus, we found that the electronic distribution in the vicinity of the Δ^7,8^ is important for activity and also we found that the most active compound **1** possessed a differential big positive zone in the MEP which could contribute to its high antifungal activity. Regarding the influence of the differential hydrophobocity of compounds in the antifungal behavior, results showed that variations in Log*P* values were not reflected in a concomitant variation of activity.
